# Potent Probiotic Yeast *Saccharomyces cerevisiae* TBRC 3616: Production Development for Food and Feed Applications

**DOI:** 10.1021/acsomega.5c11536

**Published:** 2026-03-12

**Authors:** Sompot Antimanon, Nakul Rattanaphan, Rujirek Nopgason, Thanaporn Dechpreechakul, Warinthon Chamkhuy, Yutthana Kingcha, Sasitorn Jindamorakot, Somjit Am-in, Sukitaya Veeranondha, Krith Chokpipatpol, Kobkul Laoteng

**Affiliations:** † Industrial Bioprocess Technology Research Team, Functional Ingredients and Food Innovation Research Group, National Center for Genetic Engineering and Biotechnology (BIOTEC), 61191 National Science and Technology Development Agency (NSTDA), Pathum Thani 12120, Thailand; ‡ Food Biotechnology Research Team, Functional Ingredients and Food Innovation Research Group, National Center for Genetic Engineering and Biotechnology (BIOTEC), National Science and Technology Development Agency (NSTDA), Pathum Thani 12120, Thailand; § Microbial Diversity and Utilization Research Team, Thailand Bioresource Research Center, National Center for Genetic Engineering and Biotechnology (BIOTEC), National Science and Technology Development Agency (NSTDA), Pathum Thani 12120, Thailand; ∥ Biocontrol Technology Research Team, Integrative Crop Biotechnology and Management Research Group, National Center for Genetic Engineering and Biotechnology (BIOTEC), National Science and Technology Development Agency (NSTDA), Pathum Thani 12120, Thailand; ⊥ Asia Star Trade, Bangkok 10400, Thailand

## Abstract

The global demand for probiotics has been increasing over the past few decades. Of these, *Saccharomyces cerevisiae* has attracted growing interest for use in functional foods and feed supplements due to its probiotic potential, nutritional value, and well-documented safety. For industrial applications, functional characterization, safety assessment, and robust production processes are key prerequisites. This study evaluated the probiotic properties and production potential of the *S. cerevisiae* strain TBRC 3616, isolated from decaying leaves in a tropical ecosystem in Thailand. The strain exhibited key probiotic traits, including acid and bile salt tolerance, Caco-2 cell adhesion, antipathogen activity, antioxidant capacity, and enzyme activities (catalase, protease, and esterase). The yeast exhibited no hemolytic activity and was not susceptible to the tested antibiotics, except colistin at 50 μg. High-cell-density cultivation was achieved using the developed fed-batch fermentation, resulting in a high yeast titer of 12.14 ± 0.03 log CFU L^–1^, and a cell production rate of 10.52 ± 0.02 log CFU L^–1^ h^–1^. Furthermore, downstream processing efficiency was markedly enhanced by implementing an optimized freeze-drying protocol using 5% (w/v) maltodextrin, resulting in a 4-fold increase in cell viability compared to the control. These findings provide a production framework that supports the potential for scale-up of *S. cerevisiae* TBRC 3616 as a probiotic yeast for future applications.

## Graphical abstract

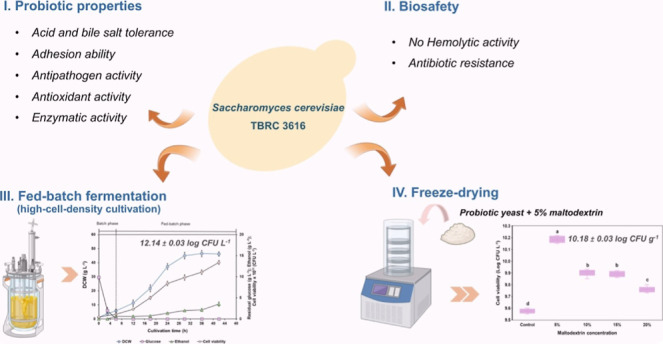

## 1 Introduction

Probiotics are defined as live, nonpathogenic microorganisms that confer health benefits to the host when administered in adequate amounts.^1,2^ Therefore, characterizing the probiotic properties is a critical step toward demonstrating their potential health benefits. Survival in the gastrointestinal tract must be addressed, including tolerance to low pH and bile salts commonly encountered in the upper digestive tract. Adhesion to intestinal epithelial cells and maintenance of viability under gastrointestinal conditions are also vital for optimal functionality in promoting gut health. Besides, antioxidant activity mitigates oxidative stress by scavenging reactive oxygen species (ROS) and reducing oxidative damage in the host. Furthermore, the antimicrobial activity against pathogenic bacteria is an essential consideration, as it plays a significant role in combating foodborne pathogens. Although antibiotics have long been used as therapeutic and prophylactic agents to prevent colonization and invasive infections, their overuse can alter intestinal microbiota and increase antibiotic resistance, thereby increasing host susceptibility to foodborne pathogens.^3–5^ Alternatively, the probiotics offer a promising approach to inhibit pathogenic bacteria and reduce the incidence of various diseases in humans and animals.^6^ From these probiotic properties, their beneficial effects are mediated through several key mechanisms: (i) enhancement of the intestinal barrier by stimulating mucin protein synthesis; (ii) modulation of the host immune system via upregulation of anti-inflammatory cytokines, interaction with intestinal epithelial cells, and recruitment of macrophages and mononuclear cells; and (iii) competitive exclusion of pathogens through the production of inhibitory metabolites, including short-chain fatty acids (SCFAs), bacteriocins, organic acids, and hydrogen peroxide; and (iv) maintenance of gut microbiota health by increasing microbial richness and diversity, enhancing enzyme (e.g., lactase) production, improving the immune microenvironment, and reducing intestinal permeability.^7–9^ Owing to these multifaceted benefits, probiotics have garnered significant interest as functional food and feed supplements over the past few decades. The global probiotics market in the food industry is projected to reach 132.51 billion USD by 2030, with a compound annual growth rate (CAGR) of 7.52–8.43% for the period between 2025 and 2530.^10–12^ Indeed, the screening and characterization of microorganisms with potential for probiotic use have become a significant focus of recent scientific progress.^13–15^ Besides strain specificity, probiotic functionality is also dose-dependent, with sufficient viable cell numbers being necessary to achieve the intended effects. In general, a daily intake of 10^7^ to 10^9^ colony-forming units (CFU) of probiotics has been reported as an effective dose.^16,17^ However, maintaining cell viability and functional properties throughout the production process remains challenging, as probiotics can lose their viability during upstream and downstream processing and storage. Additionally, cost-effective production strategies that ensure efficient large-scale manufacturing while maintaining product quality and adhering to regulatory compliance are essential.^18–20^

Several genera of microorganisms have been identified as probiotics, including *Lactobacillus*, *Bifidobacterium*, *Bacillus*, *Streptococcus*, *Enterococcus*, and *Saccharomyces*.^16,21^ Each probiotic strain exhibits distinct functional properties. For example, *Lactococcus lactis* has been reported to enhance immune function and alleviate inflammatory bowel disease, whereas *Bifidobacterium thermophilum* has demonstrated bacteriocin-like antimicrobial activity against various pathogens, including *Listeria* spp., *Salmonella* spp., *Campylobacter jejuni* in broilers, and rotavirus.^22,23^ Among these, probiotic yeasts (*Saccharomyces cerevisiae* and *Saccharomyces boulardii*) are increasingly attracting attention as alternatives or complements to bacterial probiotics in human and livestock applications. Unlike several bacterial strains, yeast probiotics exhibit superior stability during feed processing, including high-temperature pelleting, and are less sensitive to environmental stresses such as pH and oxygen fluctuations. In addition, yeast strains are inherently resistant to antibiotics commonly used in livestock, reducing the risk of probiotic inactivation during treatments. Moreover, larger yeast cells (approximately 10-fold) than bacteria allow them to exhibit steric hindrance against bacteria.^24^ These properties make yeast probiotics particularly suitable for inclusion in food and feed formulations to promote the host’s health, highlighting their practical advantages over bacterial counterparts.^1,25,26^

While individual probiotic-related traits have been documented in several yeast strains isolated from fermented foods and animal hosts, the functional probiotic properties and growth traits of plant-associated yeast strains originating from a tropical ecosystem have not been previously described. Therefore, this study aims to evaluate the probiotic properties of *S. cerevisiae* TBRC 3616 and to develop a production process for high-yield viable cell production via submerged fermentation in a stirred-tank bioreactor. Furthermore, downstream processing efficiency was enhanced by implementing an optimized freeze-drying protocol. Accordingly, this study reports the first probiotic characterization of *S. cerevisiae* TBRC 3616, a naturally derived, plant-associated yeast from a tropical ecosystem, together with a preliminary safety assessment and an evaluation of its cell production potential, thereby providing a foundation for its future development as a candidate probiotic yeast for industrial applications.

## 2 Materials and Methods

### 2.1 Microorganisms and Cultivation

*S. cerevisiae* TBRC 3616 (Thailand Bioresource Research Center; previously coded as BCC59874) was isolated from a decayed leaves collected from Doi Saket district, Chiang Mai, Thailand, and used throughout this study. *Lactobacillus plantarum* NCIMB 8826 was used as a positive control to assess probiotic properties. The yeast *S. cerevisiae* TBRC 3616 was cultivated in a 250 mL baffled flask containing 100 mL yeast peptone dextrose (YPD) medium (Difco, Le Pont de Claix, France). The cultures were incubated at 30 °C with shaking at 200 rpm for 8–12 h. Yeast cells were harvested by centrifugation at 4000*g* for 10 min and washed three times with sterile distilled (DI) water. A cell solution was used for evaluating the probiotic properties and biosafety. *L. plantarum* NCIMB 8826 was cultivated in MRS broth (Difco, Le Pont de Claix, France) at 37 °C under anaerobic conditions for 48 h, and then harvested by centrifugation at 5000*g* for 5 min. After washing the pellet, bacterial cells were resuspended in phosphate-buffered saline (PBS) (Merck, Darmstadt, Germany) and used for the evaluation.

### 2.2 Probiotic Properties Assessment

#### 2.2.1 Acid and Bile Salt Tolerance

An initial cell concentration of 10^6^ CFU mL^–1^ was inoculated into yeast extract-malt extract broth (YMB) (Difco, Le Pont de Claix, France) adjusted to pH 1.5 and 2.0 with 3 M HCl. To mimic human gastric conditions, cultures were incubated at 37 °C for 3 h, corresponding to typical stomach pH and average gastric residence time.^27,28^ Subsequently, colony counts were performed by plating the culture sample on yeast extract-malt extract agar (YM agar) (Difco, Le Pont de Claix, France) and incubating for 48–72 h at 37 °C. The number of residual viable cells was counted and expressed as CFU. The survival rate (%) of microbial cells was calculated using eq 1.

1 $$survival\;rate\;\left(\%\right)=\frac{\mathrm{Log}\;CFUN_{1}}{\mathrm{Log}\;CFUN_{0}}\times 100$$

where *N*_0_ is the number of viable cells at 0 h, and *N*_1_ is the number of viable cells after incubation under acidic and high-temperature conditions.

To assess bile salt tolerance, an initial cell concentration of 10^6^ CFU mL^–1^ was inoculated into YMB containing bile salts (Difco, Oxgall) at final concentrations of 0.5% and 1.0% (w/v). Cultures were incubated at 37 °C for 72 h. After incubation, samples were plated on YM agar using the standard colony plate count method and incubated for 48–72 h at 37 °C. Viable cells were counted, and the survival rate (%) was calculated according to eq 1.

#### 2.2.2 Antibacterial Activity against Foodborne Pathogens

The antibacterial activity of *S. cerevisiae* was evaluated against *Escherichia coli* O157:H7, *Salmonella typhimurium* ATCC 1331, and *Salmonella enterica* subsp. *enterica* ATCC 14028 using the double agar layer method modified from a previous report.^29^ Yeast culture was prepared by cultivation in YMB at 37 °C with shaking at 150 rpm for 48 h. Cell pellets were obtained by centrifugation at 4000*g* for 10 min at 4 °C. Cells were streaked on YM agar plates for 2 cm, which were then incubated at 37 °C for 48–72 h. Foodborne pathogen cultures grown in tryptic soy broth (TSB) (Difco, Le Pont de Claix, France) were added to yeast plates at an initial cell concentration of 10^5^ CFU mL^–1^. Plates were incubated at 37 °C for 24 h. Antibacterial activity was assessed by measuring the diameter of the inhibition zone using the previously described method.^14,30^

#### 2.2.3 Adhesion to Caco-2 Cells

Human Caucasian colon adenocarcinoma (Caco-2) cells were used to determine the adhesion properties of *S. cerevisiae* TBRC 3616 using a modified version of a previously established method.^31^ A yeast suspension of 10^7^ CFU mL^–1^ was added to a Caco-2 cell monolayer (4 × 10^4^ cells mL^–1^ per well) in a 24-well tissue culture plate. The culture was incubated at 37 °C under 5% CO_2_ and 95% relative humidity for 90 min. After washing the Caco-2 cells 10 times with PBS (pH 7.2), the adhered microbial cells were recovered by incubating the cells in 0.05% (w/v) Triton X-100 for 30 min, a concentration that does not damage yeast cells.^32^ The recovered cells were spread on YM agar plates and incubated at 30 °C for 24–48 h. *L. plantarum* NCIMB 8826 was employed as a positive control. The microbial colonies were counted, and the percentage of Caco-2-adhered microbial cells was calculated using eq 2.

2 $$adhesion\;\left(\%\right)=\frac{\mathrm{Log}CFU\;{mL}^{-1}\;adhesion\;microorganism}{\mathrm{Log}\;CFU{\;mL}^{-1}added\;microorganism}\times 100$$

#### 2.2.4 Enzymatic Activities

The four enzymatic activities of probiotic yeast *S. cerevisiae* TBRC 3616 were investigated individually using previously described methods,^30^ as follows:

##### 2.2.4.1 Catalase Activity

Cells were grown on a YPD plate at 37 °C for 24 h, and 30% hydrogen peroxide (H_2_O_2_) (Merck, Darmstadt, Germany) was dropped onto the colonies. The bubbles were considered a positive result.

##### 2.2.4.2 Amylase Activity

A medium was prepared with 1 L comprised of 5 g starch, 5 g peptone, 5 g yeast extract, 0.5 g MgSO_4_·7H_2_O, 0.01 g FeSO_4_·7H_2_O, 0.01 g NaCl, and 20 g agar. All chemicals used for cultivation were purchased from Carlo Erba Reagent (Val-de-Reuil, France). The medium was sterilized at 121 °C for 15 min. After 2 days of cultivation at 37 °C, the medium was flooded with iodine solution (1 L of 70 g iodine and 30 g KI; Carlo Erba Reagent, Val-de-Reuil, France). The appearance of a brown color around the colony was considered a positive result.

##### 2.2.4.3 Protease Activity

A growth medium was prepared by 1 L consisting of 10 g skim milk (Difco, Le Pont de Claix, France), 1 g glucose, 5 g peptone, 25 g yeast extract, and 20 g agar. The probiotic yeast was grown on this agar at 37 °C for 48 h. The formation of clear zones around the colonies was a positive result.

##### 2.2.4.4 Esterase Activity

To study the ability of probiotic yeast to hydrolyze ester, a growth medium that comprised 1 L of 10 g Tween 80 (polyoxyethylene-sorbitan monooleate) (Acros, Geel, Belgium), 5 g NaCl, and 0.1 g CaCl_2_·2H_2_O, 10 g peptone, and 20 g agar. The medium was adjusted to pH 6.0 and sterilized at 121 °C for 15 min. Yeast was grown on the plate at 37 °C for 48 h. The formation of clear zones around the colonies was detected as a positive result.

#### 2.2.5 Antioxidant Activity

Antioxidant activity of probiotic yeast *S. cerevisiae* TBRC 3616 was determined using the 2,2-diphenyl-1-picrylhydrazyl (DPPH) method.^33^ Yeast pellets were harvested, washed twice with PBS buffer (pH 7.4), and resuspended in 1 mL PBS. The resulting suspensions (500 μL) were combined with 1 mL of DPPH solution (0.1 mM in ethanol), vortexed, and incubated in the dark for 30 min. After that, the solutions were centrifuged at 5000*g* for 5 min, and the absorbance was measured at 517 nm. Samples prepared without yeast served as controls. The DPPH radical scavenging ability was calculated using eq 3.

3 $$scavenging\;ability\;\left(\%\right)=\frac{1-OD517\left(sample\right)}{OD517\left(control\right)}\times 100$$

### 2.3 Biosafety Evaluation

#### 2.3.1 Hemolytic Activity

The hemolytic activity of *S. cerevisiae* TBRC 3616 was investigated using the method described in a previous study.^34^ Overnight yeast cultures in YMB were streaked on 5% sheep blood agar plates (Biomedia Holding, Singapore) and incubated at 37 °C for 48 h to assess hemolysis pattern. Cultured plates were examined for a hemolytic zone, and the presence of a clear zone of hydrolysis around colonies was considered a positive result (β-hemolysis).

#### 2.3.2 Antibiotic Susceptibility Assessment

Antimicrobial resistance of *S. cerevisiae* TBRC 3616 was performed using the agar disk diffusion method as described in a previous study.^35^ Yeast suspension with a turbidity equivalent to McFarland Standard 0.5 was spread on a YM agar plate. Seven antibiotics at different concentrations, including amoxicillin (2, 10, and 25 μg), ampicillin (2, 10, and 25 μg), ciprofloxacin (1, 5, and 10 μg), colistin (10, 25, and 50 μg), erythromycin (5, 10, 15, and 30 μg), penicillin-G (1, 2, 5, and 10 μg), and tetracycline (10 and 30 μg), were applied onto sterile filter paper discs and placed on the plates. After 48 h of incubation at 37 °C, inhibition zones were observed.

### 2.4 Yeast Probiotic Production in a Bioreactor

Fed-batch fermentation of *S. cerevisiae* TBRC 3616 was carried out in a 5 L stirred-tank bioreactor (BIOSTAT B plus, Sartorius, Germany). A primary inoculum was prepared by culturing in a 250 mL Erlenmeyer flask containing 150 mL YPD medium at 30 °C with shaking at 200 rpm for 12–16 h. A secondary inoculum was prepared by transferring 5% (v/v) of the primary inoculum into synthetic medium and cultured under the same conditions for 16–18 h. The resulting culture was then transferred into 2 L of synthetic medium at an initial OD_600_ of 1.0. The synthetic medium was prepared accordingly, consisting of 10 g glucose, 4 g (NH_4_)_2_SO_4_, 13 g KH_2_PO_4_, 10 g MgSO_4_·7H_2_O, and 3 mL of trace element solution (TES) in 1 L. The TES in 1 L was composed of 6 g CuSO_4_·5H_2_O, 0.2 g Na_2_MoO_4_, 3 g MnSO_4_·H_2_O, 0.5 g CoCl_2_·6H_2_O, 15 g ZnCl_2_, and 6 g FeSO_4_·7H_2_O. All chemicals used for cultivation were purchased from Carlo Erba Reagent (Val-de-Reuil, France). The fermentation was carried out at 30 °C, and pH 4.0, adjusted with 2 M KOH or 2 M HCl. Dissolved oxygen tension (DOT) was monitored using an electrochemical sensor and calibrated under cultivation conditions by sparging nitrogen gas to define 0% oxygen and air to define 100% oxygen saturation. During fermentation, DOT was maintained at or above 20% saturation through cascade control of agitation (400–800 rpm) and aeration (1.0–1.5 vvm). Fed-batch fermentation was conducted after glucose was completely consumed, as indicated by a dramatic increase in DOT and a residual glucose concentration. The feeding medium (400 g L^–1^ glucose, 10 g L^–1^ (NH_4_)_2_SO_4_, and 2 mL L^–1^ trace element solution) was fed into a bioreactor, starting with an exponential feeding mode, followed by constant feeding at 7 mL L^–1^ h^–1^. The feed rate profile at different time points (*F*(*t*)) for exponentially increasing biomass was calculated using eq 4 based on the data from the batch stage.

4 $$F_{\left(t\right)}=\frac{\mu_{set}X_{0}V_{0}e^{\mu_{set}t}}{{S_{i}Y}_{x/s}}$$

where the μ_set_ is a maximum specific growth rate (0.2 h^–1^), *X*_0_ is the cell concentration at the time of feeding start (5.72 g L^–1^), *V*_0_ is the fermentation volume at the time of feeding start (2 L), *S*_*i*_is the substrate concentration of the feeding medium (400 g L^–1^), *Y*_*x*/*s*_ is the biomass yield on substrate (0.43 g_DCW_ g_glucose_^–1^), and *t* is the time of the fed-batch phase.

### 2.5 Optimization of Freeze-Drying Process

A freeze-drying technique was investigated for yeast cell encapsulation and drying. The types and concentrations of selected cryoprotectants were evaluated to enhance cell viability. Typically, disaccharides (sucrose, lactose, and trehalose), glycerol, skimmed milk, and maltodextrin are employed to protect probiotic cells from low temperatures.^36,37^ In this work, cost-effectiveness and high protective efficacy at low concentrations were the primary criteria for selecting a cryoprotectant. The culture broth obtained from the fermentation in a bioreactor (Section 2.4) was centrifuged at 5000*g* for 10 min at 4 °C. Cell pellets were washed three times with sterile deionized (DI) water and resuspended separately in cryoprotective solutions, including 10% (w/v) maltodextrin (Himedia, Mumbai, India), 10% (w/v) skimmed milk (Difco, Le Pont de Claix, France), or 10% (w/v) sucrose (Merck, Darmstadt, Germany) before freeze-drying. A control experiment was conducted without a cryoprotectant. Subsequently, 10 mL of each cell suspension was frozen by rolling in an ethanol bath at −20 °C, followed by freeze-drying (LyoAlfa 15, Telstar, Spain) for 24 h at −40 °C to −30 °C and 0.2 mbar. The resulting yeast probiotic powders were subjected to a cell viability test by rehydrating in sterile DI water and plating on YM agar. After incubation at 37 °C for 24 h, the colonies were enumerated and expressed as CFU g^–1^. To further investigate maltodextrin concentrations, cells were prepared under the same conditions and resuspended in maltodextrin solutions at concentrations of 5, 10, 15, and 20% (w/v). The suspensions were freeze-dried as described above, and the resulting powders were rehydrated and analyzed for cell viability.

### 2.6 Analytical Procedures

#### 2.6.1 Cell Concentration

Cell concentration in fermented culture was determined by measuring the optical density (OD) at 600 nm in a spectrophotometer (Libra S6, Biochrom Ltd., UK). Dry cell weight (DCW) was calculated by correlating OD_600_ with DCW. For DCW determination, 2 mL of the fermented culture was centrifuged at 4000*g* for 10 min. Cells were washed with DI water and dried at 60 °C in a hot-air oven until constant weight. The standard curve between DCW and OD_600_ is shown in Supporting Information Figure S1.

#### 2.6.2 Determination of Residual Glucose, Ethanol, and Organic Acid

Residual glucose, ethanol, and organic acid concentrations in the yeast culture were quantified by ultrahigh-performance liquid chromatography (UHPLC) (Ultimate 3000, Thermo Fisher Scientific, USA), which can be operated at high pressure, equipped with a refractive index detector (Refractomax 520; Dataapex). The analysis employed a 300 × 7.8 mm Aminex HPX-87H (Aminex) column. The column temperature was controlled at 60 °C. The cultured samples were prepared by filtration through a 0.2 μm cellulose acetate membrane, and 10 μL was injected. Sulfuric acid solution (5 mM) was used as a mobile phase with a flow rate of 0.6 mL min^–1^. The standard curves for residual glucose, ethanol, and acetic acid are shown in Supporting Information Figures S2–S4, respectively.

#### 2.6.3 Total Phenolic Contents (TPC)

The total phenolic contents (TPC) of culture supernatant was measured using the Folin-Ciocalteu reagent method described by Ozturk et al.^38^ In brief, 0.5 mL of sample was mixed with 0.5 mL of 0.2 N Folin–Ciocalteu reagent (Sigma, Steinheim, Germany). After incubation in the dark for 10 min, 0.6 mL of 20% (w/v) Na_2_CO_3_ (Sigma, Steinheim, Germany) was subsequently added to the reaction and incubated at 40 °C in the dark for 30 min. The solution was then centrifuged at 3000*g* for 5 min, and the absorbance was measured at 765 nm with a spectrophotometer. Gallic acid was used as the standard reagent, and the standard curve is presented in Supporting Information Figure S5.

#### 2.6.4 Kinetic Parameters Calculation

The kinetic parameters for probiotic yeast of *S. cerevisiae* TBRC 3616 production were determined, including specific growth rate (μ), biomass yield on substrate (*Y*_*x*/*s*_), substrate consumption rate (*Q*_s_), and biomass production rate (*Q*_*x*_).

#### 2.6.5 Statistical Analysis of Experimental Data

All values, except for antibacterial activity, antibiotic susceptibility assessment, and bioreactor experiment, represent the mean with their standard deviation (mean ± SD) of three biological replicates. The normality of residuals and homogeneity of variances were evaluated using one-way analysis of variance (ANOVA), and Tukey’s HSD test was applied to assess statistical differences using SPSS version 11.5 (SPSS software, Chicago, USA). Data were considered statistically significant at *p*-values ≤ 0.05.

## 3 Results and Discussion

### 3.1 Probiotic Properties of *S. cerevisiae* TBRC 3616

Characterization of probiotic properties represents a primary step in screening microorganisms for potential probiotic applications. The investigation of the probiotic attributes of *S. cerevisiae* TBRC 3616, including acid and bile salt tolerance, adhesion to Caco-2 cells, and enzyme, antibacterial, and antioxidant activities, showed as follows:

#### 3.1.1 Acid Tolerance

Acid tolerance is a critical characteristic of probiotic microorganisms, enabling them to survive stomach acidity (pH 1.5–3.0) and reach the intestinal tract in a viable state, thereby conferring health benefits to the host. The analyzed values of acid tolerance of *S. cerevisiae* TBRC 3616 are presented in Table 1. The strain exhibited a high survival rate of over 78% under acidic conditions. Increasing the pH from 1.5 to 2.0 significantly enhanced the survival rate by approximately 7% (*p* < 0.05). These results aligned with previous reports by Ng et al. and Kou et al., which demonstrated that decreasing pH adversely affected microbial survival.^39,40^ Moreover, Li et al. reported that low pH conditions led to reduced tolerance and decreased cell viability.^41^

**Table 1:** Survival Rate of *S. cerevisiae* TBRC 3616 under Different pHs and Bile Salt Concentrations, and Its Antibacterial Activity Against Foodborne Pathogens^a^

acid and bile salt tolerance		
parameters	survival rate (%)	required for probiotic status
pH 1.5	78.06 ± 1.85^b^	≥70% ^14,33^
pH 2.0	83.38 ± 1.05^a^	
0.5% Bile salt	78.65 ± 1.75^a^	
1.0% Bile salt	78.56 ± 1.46^a^	

antibacterial activity	
pathogenic bacteria	inhibition zone (mm)
*E. coli* O157:H7	7.20 ± 0.35
*S. typhimurium* ATCC 1331	0.00 ± 0.00
*S. enterica subsp. enterica* ATCC 14028	8.14 ± 1.20

a Note: Data on acid and bile salt tolerance are given as means ± SD (*n* = 3). Values marked with different superscript letters within the same column for each factor indicate significantly different (*p* < 0.05).

#### 3.1.2 Bile Salts Tolerance

The result of bile salt tolerance at concentrations of 0.5–1.0% (w/v) under simulated body temperature (37 °C) is shown in Table 1. The survival rate of *S. cerevisiae* TBRC 3616 was about 78% in the presence of 0.5% and 1.0% bile salts. These findings indicate that the strain can survive in conditions of the small intestine, where bile salt concentrations range from 0.3% to 1.0%.^42–44^ Additionally, the average small-intestinal transit time is approximately 4 h.^45^ Therefore, *S. cerevisiae* TBRC 3616 meets the essential criteria for probiotics and was selected for further evaluation of its probiotic properties.

The observed tolerance of *S. cerevisiae* TBRC 3616 to acidic conditions and bile salts can be attributed to several intrinsic structural and physiological mechanisms characteristic of yeast probiotics. Yeast cells have a thick, highly organized cell wall composed primarily of β-glucans, mannoproteins, and chitin, which acts as a physical barrier, limiting the penetration of hydrogen ions and bile salts.^46^ This structure might reduce membrane disruption and protect intracellular components under harsh gastrointestinal conditions. In addition, exposure to acidic environments triggers adaptive stress responses in yeast, including the activation of plasma membrane H^+^-ATPases that eliminate excess protons to maintain intracellular pH homeostasis.

#### 3.1.3 Antibacterial Activity

Antibacterial activity is a key characteristic of probiotics in combating foodborne pathogens. Therefore, the beneficial effect of *S. cerevisiae* TBRC 3616 against foodborne pathogens, *E. coli* O157:H7, *S. typhimurium*, and *S. enterica* subsp. *enterica* was investigated. The results showed that *S. cerevisiae* TBRC 3616 strongly inhibited *E. coli* O157:H7 and *S. enterica* subsp. *enterica*, while exhibiting no inhibition against *S. typhimurium* (Table 1). This may primarily be attributed to the action of organic acids, especially acetic acid, which can more readily penetrate the cell membranes of *E. coli* O157:H7 and *S. enterica* than those of *S. typhimurium*, likely due to differences in membrane composition and permeability. Acetic acid is broken down inside the cell, producing hydrogen ions and anions in the cytoplasm, thereby significantly decreasing intracellular pH. This acidic stress inhibits pH-sensitive enzymes in pathogens, leading to dysfunction in energy metabolism and DNA replication.^47,48^ In addition, receptor antagonism may also contribute to inhibiting the *E. coli* O157:H7 and *S. enterica* because they show the quorum-sensing (QS) receptor (AgrC). These can be supported by organic acids, which can suppress the expression of virulence genes regulated by QS systems in pathogens, reducing their invasive capability.^48^

#### 3.1.4 Caco-2 Cell Adhesion Efficiency of *S. cerevisiae* TBRC 3616

Epithelial adhesion is a crucial characteristic of probiotics for host colonization. It is a specific interaction between microbial surface components and the complementary structure of the host cell surface in the digestive tract.^49–51^ The in vitro study of yeast cells adhering to Caco-2 cells (nonmucus-secreting) showed that strain TBRC 3616 adhered tightly to Caco-2 cells (83.20%), with no significant difference compared to the positive control, *L. plantarum* NCIMB 8826 (*p* > 0.05), as illustrated in Figure 1. The adhesion efficiency of *S. cerevisiae* TBRC 3616 to Caco-2 cells observed in this study was notably higher than values reported previously. Kil et al. documented that the maximum adhesion ability of the *S. cerevisiae* GILA strain was 15%,^52^ whereas *S. cerevisiae* YH14 exhibited adhesion of 43.18% to Caco-2 cells.^32^

**Figure 1:**
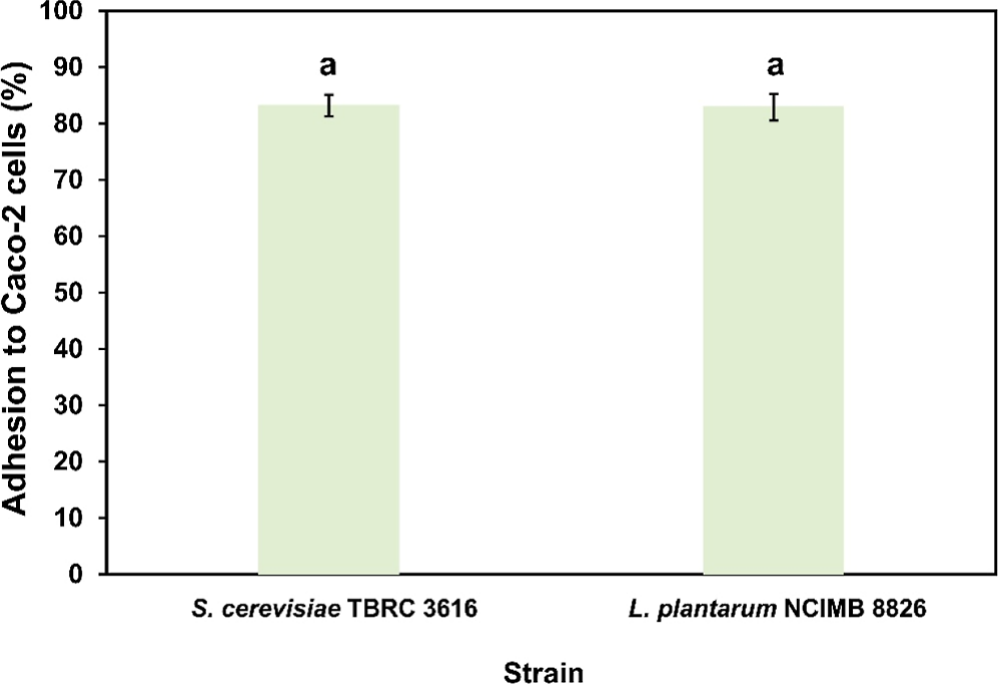
Caco-2 cell adhesion test of *S. cerevisiae* TBRC 3616 (83.20 ± 1.90%) and *L. plantarum* NCIMB 8826 (82.90 ± 2.31%). All data are presented as mean ± SD (*n* = 3). Statistical significance is indicated with different letters (*p* < 0.05).

#### 3.1.5 Enzymatic Activity of *S. cerevisiae* TBRC 3616

The enzymatic activities of *S. cerevisiae* TBRC 3616, including catalase, amylase, protease, and esterase, were evaluated. As shown in Table 2, catalase, protease, and esterase activities were detected. These enzymes are relevant to probiotic functionality, as catalase protects against oxidative stress, protease facilitates the breakdown of dietary proteins into smaller peptides and amino acids, and esterase participates in lipid metabolism by hydrolyzing esterified compounds.^30^ In contrast, no amylase activity was detected. This observation is consistent with previous reports indicating that several *S. cerevisiae* strains lack extracellular amylase activity and primarily metabolize simpler sugars.^53^ The absence of amylase activity does not diminish the probiotic potential of this yeast since starch degradation is not a core requirement for probiotic functionality and can be complemented by dietary enzymes or other members of the gut microbiota.

**Table 2:** Antioxidant and Enzymatic Activities of *S. cerevisiae* TBRC 3616^a^

probiotic yeast	antioxidant activity	TPC (mg mL^–1^)
	DPPH radical scavenging activity (%)	
*S. cerevisiae* TBRC 3616	59.96 ± 1.03	492.73 ± 7.88

enzymatic activities	result
catalase	+
amylase	-
protease	+
esterase	+

a Note: Data on DPPH radical scavenging activity and TPC are given as means ± SD (*n* = 3). The results of enzymatic activities are presented as follows: (−) negative, (+) positive.

#### 3.1.6 Antioxidant Ability of *S. cerevisiae* TBRC 3616

Antioxidant activity refers to the ability of probiotics to counteract oxidative stress by neutralizing reactive oxygen species (ROS) and reducing oxidative damage in the host. In this study, the DPPH free radical-scavenging capacity of *S. cerevisiae* TBRC 3616 was evaluated. The results indicated that *S. cerevisiae* TBRC 3616 exhibited high DPPH radical-scavenging activity (Table 2), suggesting an antioxidant capacity. This activity correlates with the observed positive catalase activity, which contributes to cellular defense against oxidative stress by decomposing hydrogen peroxide. These might enhance probiotic survival in the gastrointestinal tract and support host antioxidant balance.^30,54^ Additionally, TPC was detected at a concentration of 492.73 ± 7.88 mg mL^–1^. This compound has been documented to exhibit several beneficial properties, including antioxidant, antibacterial, and anti-inflammatory activities, which support the probiotic properties of *S. cerevisiae* TBRC 3616.

Conclusively, the yeast strain TBRC 3616 exhibited probiotic-relevant properties, including acid and bile tolerance, Caco-2 cell adhesion, enzymatic, antimicrobial, and antioxidant activities. Notably, *S. cerevisiae* TBRC 3616 originates from a tropical plant-associated ecosystem, distinct from previously reported *S. cerevisiae* strains (i.e., GILA, C41, or YH14) isolated from fermented foods or animal hosts,^32,52,55^ thereby expanding the ecological and functional diversity of candidate *S. cerevisiae* probiotic strains and providing a comprehensive basis for its potential in the food and feed industries.

### 3.2 Biosafety Assessment

Biosafety assessment of potential probiotic strains is vital since some yeast isolates may be pathogenic.^56,57^ Therefore, biosafety evaluation is necessary for all new potential probiotic strains for their application in humans and animals.^58^ In the present study, we investigated the hemolytic activity and antibiotic susceptibility of *S. cerevisiae* TBRC 3616 to preliminarily assess its safety profile for potential use as a probiotic yeast.

#### 3.2.1 Hemolytic Activity

Hemolytic activity of *S. cerevisiae* TBRC 3616 was analyzed. As shown in Figure 2, no clear zone was observed on the sheep blood agar (5%, w/w), indicating that the strain TBRC 3616 had no adverse effect on hemolysis. This result is consistent with a previous report by Romero-Luna et al., who found that the probiotic yeast *S. cerevisiae* strain (C41) was also considered safe due to the absence of hemolytic activity.^55^ In addition, *S. cerevisiae* BTS1-KO showed no hemolytic activity, indicating it was safe for human application.^59^

**Figure 2:**
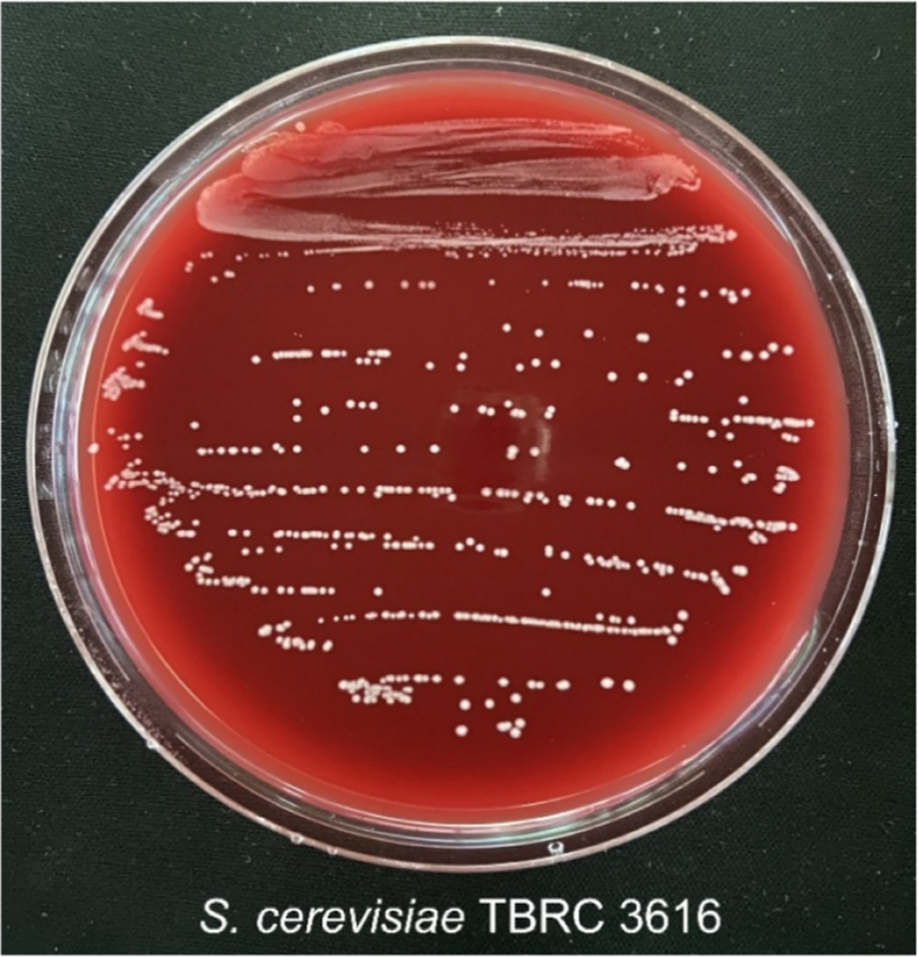
Hemolytic activity test of *S. cerevisiae* TBRC 3616 on sheep blood agar (5%, w/v).

Nevertheless, whole-genome sequencing and in silico analysis of virulence factors, toxin genes, and transferable antibiotic resistance genes are essential next steps before any practical application.

#### 3.2.2 Assessment of Antibiotic Susceptibility

A key criterion of probiotics is their resistance to antibiotics, which enables them to help restore gut microbiota after antibiotic administration.^24,30^ In addition, the European Food Safety Authority guideline addresses antibiotic susceptibility testing for use with microorganisms in food production or as additives.^60,61^ Of the strain testing, *S. cerevisiae* TBRC 3616 demonstrated resistance to all antibiotics tested at different concentrations, except colistin. As shown in Table 3, yeast TBRC 3616 was resistant to colistin at 10 and 25 μg, but was susceptible at 50 μg at body temperature. This finding is consistent with the report by Wang et al., who found that the probiotic yeast *S. cerevisiae* was not resistant to colistin at 100 μg.^24^ Several studies showed divergence in *S. cerevisiae* drug resistance, suggesting that it is strongly associated with the strain analyzed. Although antibiotic resistance genes in yeast cannot be horizontally transferred to bacteria, probiotic yeast should be sensitive to at least one drug, especially since recent medical reports have highlighted a few cases of fungemia in patients treated with *S. cerevisiae* var. *boulardii* probiotics.^28^ In the case of the *S. cerevisiae* TBRC 3616 strain, it was sensitive to colistin at 50 μg. Colistin is primarily used against Gram-negative bacteria. However, several studies have reported that colistin, especially at high concentrations or in combination with other antimicrobial agents, can inhibit the growth of eukaryotic cells, including fungi and yeasts. Although the mechanism of colistin activity in eukaryotic cells remains unclear, it has been suggested that colistin may interact with cell wall components and disrupt phospholipid membranes, leading to membrane destabilization and cellular damage.^62,63^

**Table 3:** Susceptibility to Seven Antibiotics of *S. cerevisiae* TBRC 3616^a^

antibiotics (μg)	antibiotic activity
amoxicillin (2, 10, 25)	R
ampicillin (2, 10, 25)	R
ciprofloxacin (1, 5, 10)	R
colistin (10, 25)	R
colistin (50)	S
erythromycin (5, 10, 15, 30)	R
penicillin-G (1, 2, 5, 10)	R
tetracycline (10, 30)	R

a R = Resistance; S = Susceptibility.

### 3.3 Process Development of Probiotic Yeast Production by Fed-Batch Fermentation

Using fed-batch fermentation in a 5 L stirred tank bioreactor, the yeast cells increased during the first growth stage (batch), with a DCW of 5.72 ± 1.20 g L^–1^ and viable cells of 1.11 ± 0.12 × 10^11^ CFU L^–1^ (11.05 ± 0.01 log CFU L^–1^). After 6 h of cultivation, glucose was consumed entirely, and the fed-batch stage was proceeded by adding feed medium solution using an exponential feeding strategy for 12 h. Subsequently, a constant feeding mode at 7 mL L^–1^ h^–1^ was applied to increase cell concentration further and prevent oxygen depletion and nutrient exhaustion. As shown in Figure 3, yeast cells continuously grew throughout the fed-batch phase without glucose accumulation in the culture.

**Figure 3:**
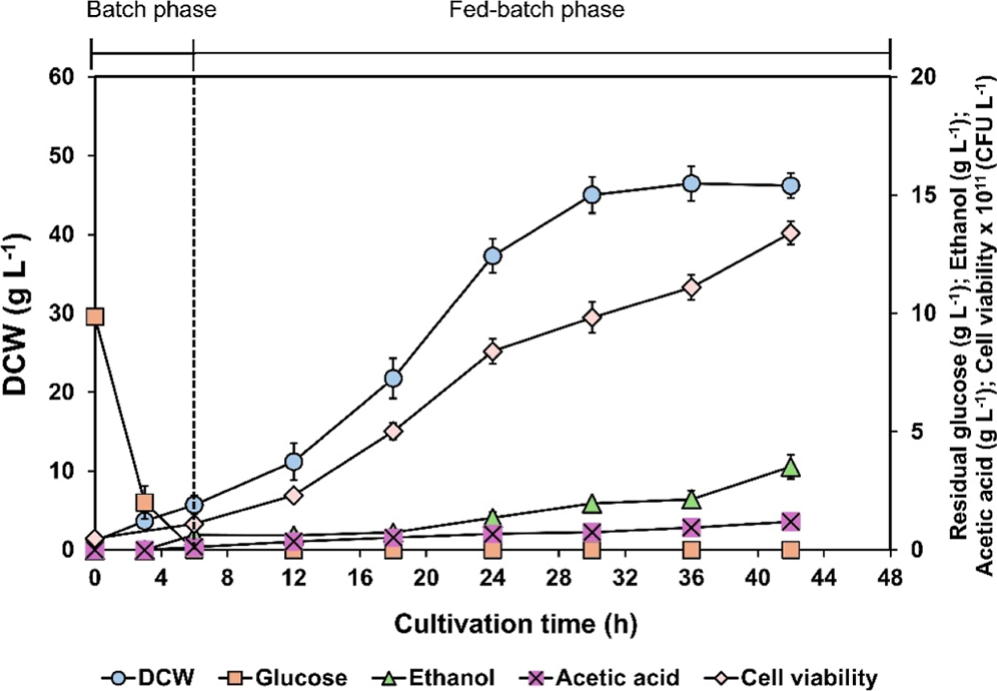
Growth profile of probiotic yeast *S. cerevisiae* TBRC 3616, residual glucose concentration, and byproduct formation (ethanol and acetic acid) during cultivation in a stirred-tank bioreactor. Data are presented as means ± standard deviation.

Although DCW plateaued at approximately 46 g L^–1^ after 30 h fermentation, cell viability dramatically increased, reaching a final of 1.40 ± 0.12 × 10^12^ CFU L^–1^ (12.14 ± 0.03 log CFU L^–1^). Ethanol and acetic acid were observed in the culture broth at a low concentration (0.63 ± 0.10 g L^–1^ and 0.12 ± 0.10 g L^–1^, respectively) during the batch phase, and then increased to 3.52 ± 0.50 g L^–1^ and 1.20 ± 0.15 g L^–1^, respectively, by the end of the fed-batch fermentation.

This might be the result of dissolved oxygen levels being controlled at 20% saturation, and *S. cerevisiae* is Crabtree-positive, meaning it can produce ethanol even when the process is operated in fully aerobic conditions due to overflow metabolism.^64–66^ However, ethanol was predominantly extracellular and present at negligible levels within the probiotic yeast cells. Furthermore, *S. cerevisiae* is inherently tolerant to ethanol at concentrations substantially higher than those observed in this study.^67^ Therefore, the observed ethanol concentration is not expected to adversely affect cell viability or pose safety concerns for food and feed applications. Indeed, this distinct ecological origin likely contributes to its unique stress–adaptation profile, particularly tolerance to environmental fluctuations, oxidative stress, and nutrient limitation, all of which are relevant to gastrointestinal survival and stress conditions encountered during high-cell-density fermentation. As a result, the kinetic parameters for cell growth, glucose consumption, and cell viability of the probiotic yeast TBRC 3616 (Table 4) were higher than those reported in other yeast strains in previous studies.^68,69^

**Table 4:** Fermentation Parameters of Probiotic *S. cerevisiae* TBRC 3616 Production Using Fed-Batch Fermentation^a^

fermentation parameters					
DCW (g L^–1^)	cell viability (log CFU L^–1^)	μ (h^–1^)	*Y*_*x*_/_*s*_ (g cell g glucose^–1^)	*Q*_*x*_ (Log CFU L^–1^ h^–1^)	*Q*_s_ (g glucose L^–1^ h^–1^)
45.20 ± 2.20	12.14 ± 0.03	0.25 ± 0.02	0.43 ± 0.03	10.52 ± 0.02	2.61 ± 0.13

a Data are expressed as means ± standard deviation.

### 3.4 Optimized Freeze-Dried Condition for Probiotic Yeast

The viable cell count of probiotic yeast in finished goods is essential for ensuring functional activity in the consumer’s gut. Although the upstream process of high-cell-density cultivation was developed to enhance cell viability and titer, the product drying is an essential step in probiotic production. There are several methods, such as spray drying, spray chilling, and fluidized bed drying, that have been employed for probiotics; they are often associated with high viable cell loss, resulting in low yields.^70,71^ In this study, we selected the freeze-drying method to produce a probiotic powder of *S. cerevisiae* TBRC 3616 by comparing the efficiency of three cryoprotectants (sucrose, skimmed milk, and maltodextrin). As shown in Figure 4a, the highest efficiency in preserving the probiotic yeast was achieved with skimmed milk, resulting in about 10.12% higher cell viability than the control. The probiotic powder containing maltodextrin maintained a cell viability of 10.08 ± 0.02 log CFU g^–1^, which was approximately 3.40% lower than that of the skimmed milk powder. Using sucrose, there was no significant effect on cell viability compared to the control (*p* > 0.05). This result coincided with the report by Cao et al., who demonstrated that the addition of a cryoprotective agent reduced *S. cerevisiae* loss, and that 11% (w/v) skimmed milk powder increased cell viability by 76.36%.^37^ Using sucrose as a cryoprotectant for *L. plantarum* freeze-drying, the cell survival rate was lowest compared to that of trehalose, skimmed milk powder, and maltodextrin.^72^

**Figure 4:**
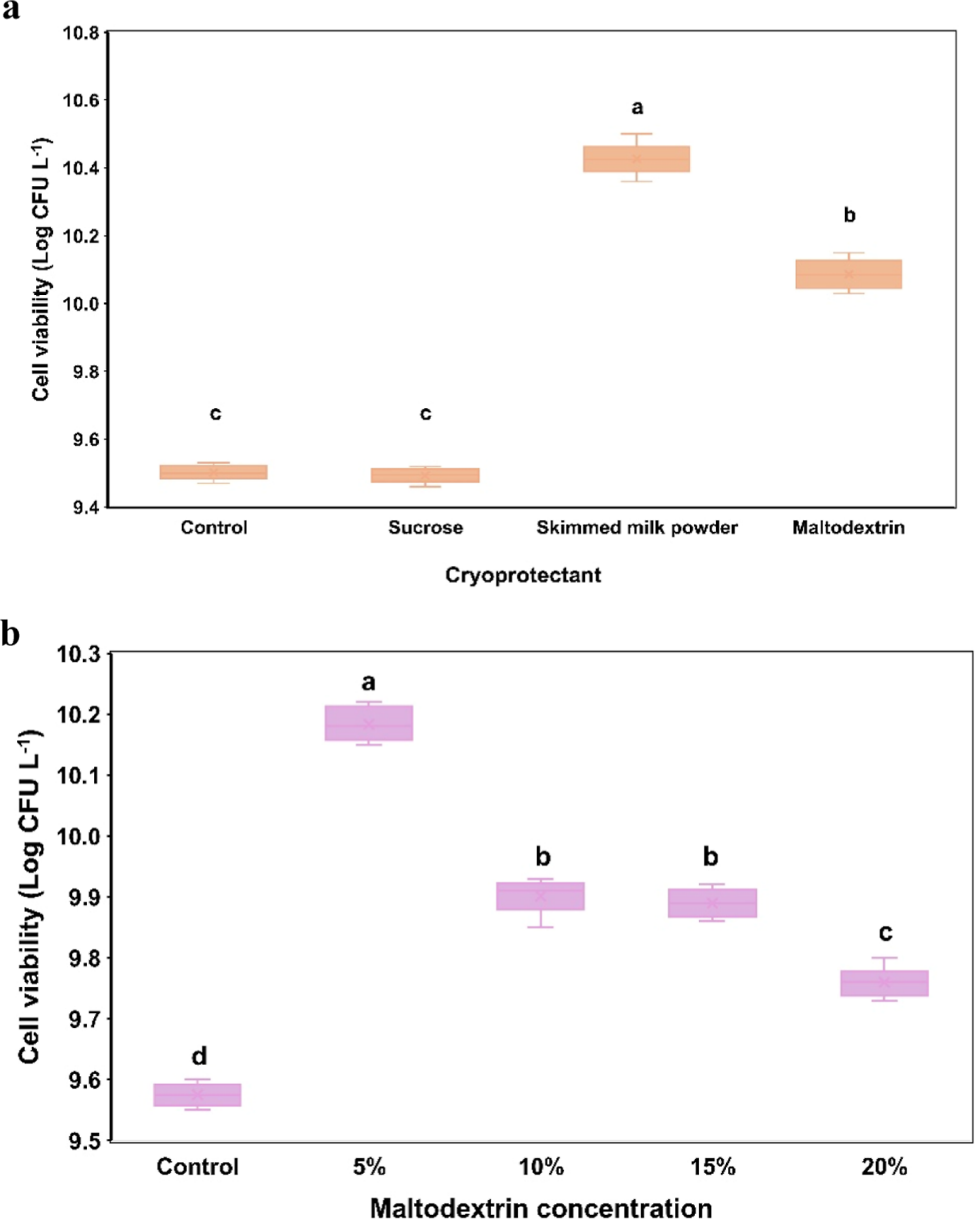
Freeze-drying optimization for probiotic yeast *S. cerevisiae* TBRC 3616. (a) The effect of various cryoprotectants on cell viability. (b) The impact of maltodextrin concentration on cell viability. All data are presented as mean ± SD (*n* = 3). Statistical significance is indicated with different letters (*p* < 0.05).

Although the maximum viable cell count was achieved with skimmed milk powder, its price is about 3.5 times that of maltodextrin by local market price, which would increase production costs on a large scale. Importantly, some consumers are allergic to milk protein. Therefore, maltodextrin was used as the cryoprotectant in subsequent experiments. Using different maltodextrin concentrations for freeze-drying of yeast cells, we found that the high maltodextrin concentration (10–20%, w/v) significantly reduced cell viability (Figure 4b). Of them, 5% (w/v) maltodextrin was the optimal concentration for efficiently preserving yeast viability, resulting in a 4-fold increase over the control, with viable cells at 10.18 ± 0.03 log CFU g^–1^, and the moisture content in the final powder was 4.32 ± 0.21%. It has been reported that trehalose, a commonly used cryoprotectant, can improve cell viability during freeze-drying, but the high concentrations required (often >15% w/v) may limit its practical use due to production costs.

## 4 Conclusion

The tropical yeast, *S. cerevisiae* TBRC 3616, exhibited probiotic properties, including acid and bile salt tolerance, Caco-2 cell adhesion, antipathogen activity, antioxidant capacity, and enzyme activity (catalase, protease, and esterase). Furthermore, the strain met preliminary safety criteria by showing no hemolytic activity and intrinsic resistance to the tested antibiotics, except colistin (50 μg). An efficient and cost-effective production process for the probiotic yeast *S. cerevisiae* TBRC 3616, encompassing both upstream and downstream processing, was successfully developed, providing a robust foundation for further scale-up. The techno-economic analysis and functional validation at pilot-scale production of the probiotic yeast, along with intensive safety evaluation through whole-genome sequencing and assessment of advanced probiotic attributes (e.g., cholesterol assimilation), could provide critical insights into industrial feasibility and regulatory considerations. These efforts would aid in translating the present findings into practical applications and strengthening the commercial potential of *S. cerevisiae* TBRC 3616.

## Supplementary Material



## Notes

Standard curve relating OD_600_ to dry cell weight (DCW) of *S. cerevisiae* TBRC 3616 (Figure S1). Standard curves of glucose concentration (Figure S2), ethanol concentration (Figure S3), acetic acid concentration (Figure S4), and gallic acid concentration (Figure S5) analyzed by HPLC (PDF)
