# Supplementary material for: Potent Probiotic Yeast Saccharomyces cerevisiae TBRC 3616: Production Development for Food and Feed Applications
Source: ACS Omega. 2026 Mar 12;11(11):17652–62. doi: 10.1021/acsomega.5c11536 (PMC13019213; doi:10.1021/acsomega.5c11536)
Supplement: Supplementary file 1 [file ao5c11536_si_001.pdf]

Supporting Information

Potent probiotic yeast *Saccharomyces cerevisiae* TBRC 3616: Production development for food and feed applications

Sompot Antimanon[1], Nakul Rattanaphan[1], Rujirek Nopgason[1], Thanaporn Dechpreechakul[1], Warinthon Chamkhuy[1], Yutthana Kingcha[2], Sasitorn Jindamorakot[3], Somjit Am-in[3], Sukitaya Veeranondha[4], Krith Chokpipatpol[5], Kobkul Laoteng[1]*

[1]Industrial Bioprocess Technology Research Team, Functional Ingredients and Food Innovation Research Group, National Center for Genetic Engineering and Biotechnology (BIOTEC), National Science and Technology Development Agency (NSTDA), Pathum Thani 12120, Thailand

[2]Food Biotechnology Research Team, Functional Ingredients and Food Innovation Research Group, National Center for Genetic Engineering and Biotechnology (BIOTEC), National Science and Technology Development Agency (NSTDA), Pathum Thani 12120, Thailand

[3]Microbial Diversity and Utilization Research Team, Thailand Bioresource Research Center, National Center for Genetic Engineering and Biotechnology (BIOTEC), National Science and Technology Development Agency (NSTDA), Pathum Thani 12120, Thailand

[4]Biocontrol Technology Research Team, Integrative Crop Biotechnology and Management Research Group, National Center for Genetic Engineering and Biotechnology (BIOTEC), National Science and Technology Development Agency (NSTDA), Pathum Thani 12120, Thailand

[5]Asia Star Trade, Bangkok 10400, Thailand

*Correspondence: Kobkul Laoteng. Email: kobkul@biotec.or.th

This file includes:

Figure S1 to Figure S5

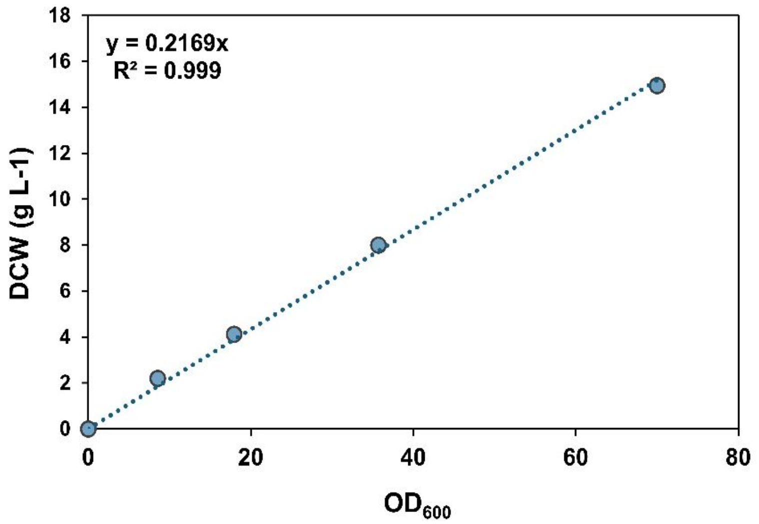

Figure S1. Standard curve between OD_{600} and dry cell weight (DCW) of *S. cerevisiae* TBRC 3616

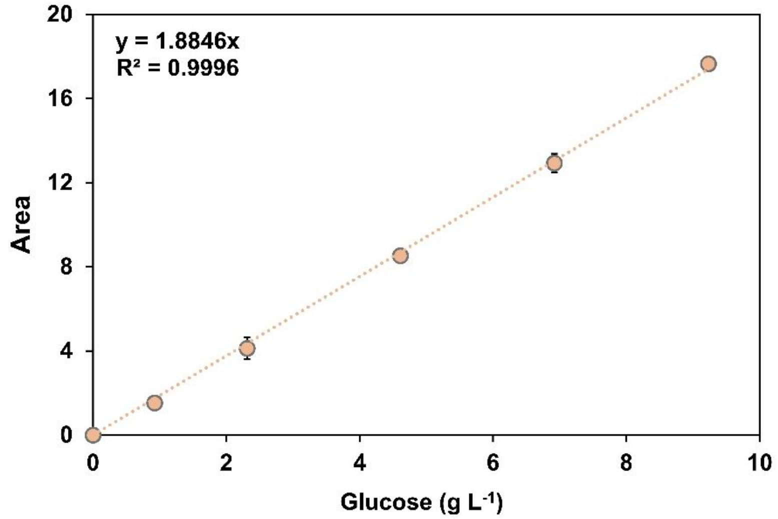

Figure S2. Standard curve of glucose concentration analyzed by HPLC

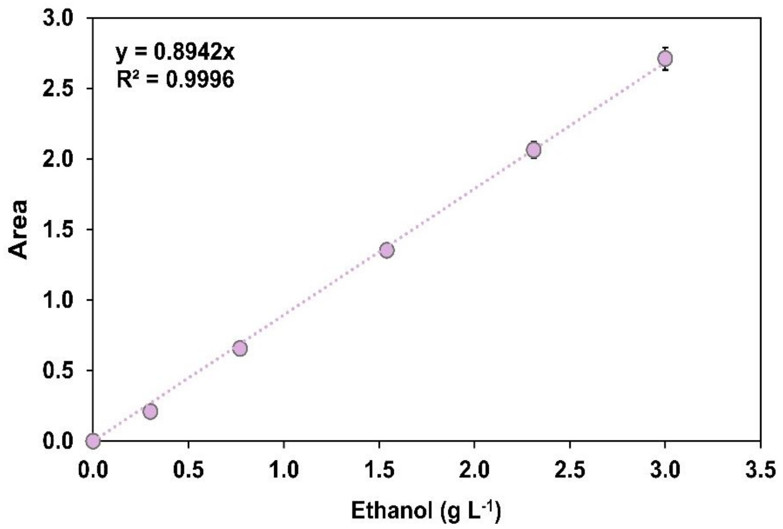

Figure S3. Standard curve of ethanol concentration analyzed by HPLC

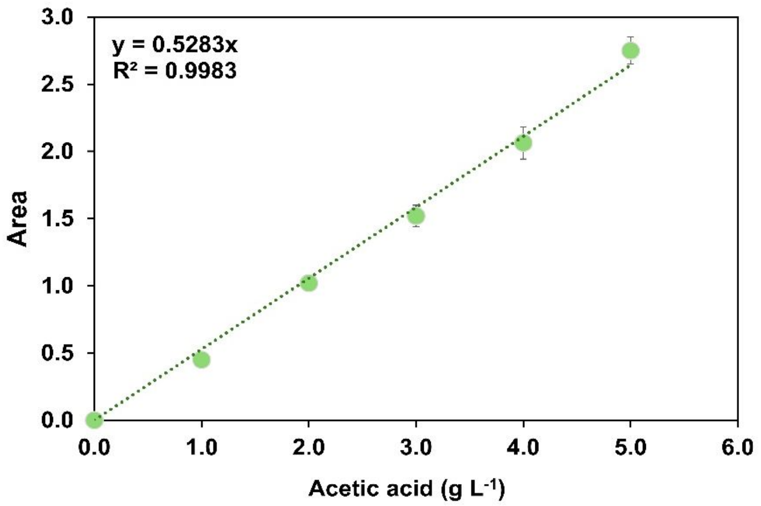

Figure S4. Standard curve of acetic acid concentration analyzed by HPLC

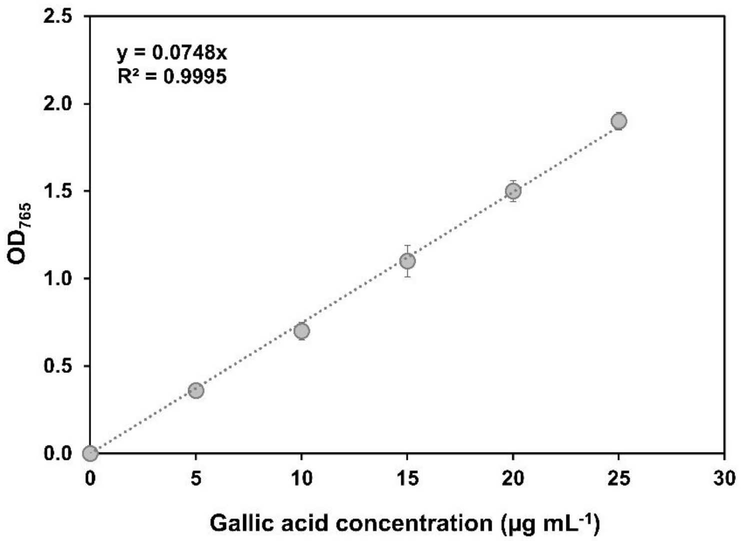

Figure S5. Standard curve of gallic acid concentration
